# A Lifelong Journey in the Field of the Pulmonary Circulation: Perspectives Through the Lens of the Cardiovascular Pulmonary Research Laboratory

**DOI:** 10.1002/pul2.70222

**Published:** 2025-12-10

**Authors:** Kurt R. Stenmark

**Affiliations:** ^1^ University of Colorado Anschutz Aurora Colorado USA

In 1980, while working as a pediatric resident in the newborn intensive care unit of a University‐affiliated hospital, I was called urgently to the delivery room. The attending obstetrician had delivered a full‐term infant without complications just minutes before my arrival. The baby, however, was cyanotic and in severe respiratory distress. I administered oxygen via hand‐bagging, but the infant's color and condition failed to improve. I intubated the child, continued assisted ventilation, and called the attending physician at the University of Colorado Hospital. During our brief but critical discussion, we concluded that the newborn likely suffered from persistent pulmonary hypertension of the newborn (PPHN)—a condition in which the pulmonary arteries remain constricted after birth, preventing proper oxygenation.

At the time, this was a devastating diagnosis with no effective therapies. The infant was transferred to the University Hospital, where IV Priscoline was administered along with continued ventilation. Despite every effort, the baby passed away within hours. The parents—both fellow residents—were heartbroken, as was I. My grief turned into determination. I resolved that I would dedicate my career to understanding this disease—no matter how long it might take.

A few years later, that determination led me to a fateful introduction. Dr. Ernest “Ernie” Cotton, director of the Pediatric Pulmonary Fellowship at the University of Colorado, introduced me to Dr. Jack Reeves—one of the world's leading experts in pulmonary hypertension (PH) and co‐director of the Cardiovascular Pulmonary (CVP) Research Laboratories. Our first meeting convinced me that, under his mentorship and within the collaborative environment of the CVP, we could begin to uncover the mechanisms and potential treatments for PPHN.

## 1 The Beginning of the Cardiovascular Pulmonary Research (CVP) Lab

The CVP was founded in 1965 as an extension of the renowned Cardiothoracic Research Center, led by the pioneering investigators Henry Swan and Gilbert Blount [1]. Drs. Robert Grover and Jack Reeves established the CVP to investigate the pathogenesis of pulmonary hypertension (PH) at high altitudes—where oxygen tension is reduced. Their studies of residents living in Leadville, Colorado, and of native high‐altitude populations in Peru, Bolivia, and Nepal provided critical insights into hypoxia‐induced PH.

They also investigated “brisket disease,” a form of PH affecting cattle at high altitudes—a major problem for Colorado ranchers. Working with pathologists Drs. Alexander and Will, Reeves and Grover performed landmark experiments showing that the severity of right ventricular (RV) hypertrophy was directly proportional to mean pulmonary artery pressure (PAP) [2]. Delivering 100% oxygen lowered PAP, but not to normal levels, suggesting lasting vascular changes.

These studies revealed that chronic hypoxia led to progressive vascular remodeling, including smooth muscle hypertrophy and increased resistance, laying the foundation for our understanding of hypoxic PH [2, 3, 4, 5, 6, 7]. These pioneering observations were featured at the first World Health Organization (WHO) Congress on Pulmonary Hypertension in Geneva in 1973, attended by Dr. Blount [8], and inspired generations of PH research to follow.

Throughout the 1970s, the CVP grew into a dynamic hub of investigation, recruiting talented scientists such as Alan Tucker, Ivan McMurtry, Ken Weir, and Lorna Moore. Together, they explored exaggerated hypoxic responses, vascular smooth muscle function, and genetic predisposition to high‐altitude PH in cattle [9, 10], as well as mechanisms of hypoxic vasoconstriction [11, 12, 13, 14]. They were among the first to demonstrate that calcium antagonists like verapamil could inhibit hypoxic vasoconstriction—a major therapeutic insight.

## 2 My Initial Hypothesis

When I joined the CVP in early 1982, the laboratory was deeply engaged in exploring mechanisms of pulmonary vascular reactivity and injury. Dr. Norbert Voelkel, a new addition to the team, was examining the role of prostaglandins and arachidonic acid metabolites in regulating vascular tone [15, 16]. He and his collaborators, including Dr. Robert C. Murphy in Pharmacology, were particularly focused on leukotrienes, newly recognized lipid mediators with potent effects on the pulmonary circulation [17, 18, 19].

On my very first day, Jack Reeves tasked me with reviewing all existing literature on pulmonary vascular disease and PPHN—and then presenting a hypothesis to him. That meant long hours in the library, sifting through bound journals and microfilm archives. Drawing from discussions with CVP researchers and colleagues like Richard Johnson and Peter Henson at National Jewish Hospital, I proposed that leukotrienes (particularly LTC₄), induced under hypoxic conditions, might drive key features of PPHN—pulmonary vasoconstriction, bronchoconstriction, reduced compliance, and edema.

After months of work, our team demonstrated, for the first time in humans, elevated levels of leukotrienes C₄ and D₄ in the bronchoalveolar lavage (BAL) fluid of infants with PPHN—but not in ventilated infants without PPHN [20]. This discovery ignited a wave of interest in leukotrienes and their role in pulmonary hypertension across the lifespan.

To further probe disease mechanisms, we needed a robust animal model. Building on the earlier bovine work by Tucker, McMurtry, and Reeves, we hypothesized that newborn calves exposed to hypoxia shortly after birth might recapitulate human PPHN. When 2‐day‐old calves were placed at simulated high altitude (4300 m), they developed supra‐systemic pulmonary pressures that persisted even with 100% oxygen [21]. Pathological examination revealed marked adventitial thickening, smooth muscle hypertrophy, and distal muscularization, closely mirroring human PPHN.

Interestingly, while we did not find leukotriene abnormalities as the main driver, we discovered a striking reduction in prostaglandin production—a finding that foreshadowed the eventual development of prostacyclin therapy [22]. Indeed, by 1995, Epoprostenol became the first FDA‐approved drug for PH [23].

Throughout the 1980s, our team leveraged the neonatal calf model to dissect endothelial and smooth muscle dysfunction, extracellular matrix remodeling, and biomechanical consequences of vascular stiffening [24, 25, 26, 27, 28, 29, 30]. We uncovered distinct smooth muscle subpopulations and demonstrated that ACE activity drives muscularization of small arteries [31]. Collaborations with bioengineers, including Drs. Shandas, Hunter, and Tan, revealed that changes in connective tissue and vessel stiffness profoundly influence right ventricular load and failure [32, 33, 34]. These insights opened new avenues for targeting vascular stiffness as a therapeutic goal [35].

## 3 Building the CVP Into a Translational Powerhouse

In 1990, the NIH launched the Specialized Center of Research (SCOR) program to support major investigations in cardiovascular disease. Together with Jack Reeves, I assembled a multidisciplinary team—including Stephen Abman, Carl White, John Kinsella, and Dunbar Ivy—to study pulmonary hypertension in infants. Our program, “SCOR—Lung Biology and Diseases in Infants and Children,” was funded for 20 years and produced transformative discoveries.

One of these was the development of inhaled nitric oxide (iNO) therapy, which revolutionized the treatment of neonatal PH and remains the global standard of care [36, 37, 38, 39].

## 4 Expanding Horizons: Cellular Diversity and Inflammation

That same year, I attended a US–USSR symposium on cardiovascular biology and met a brilliant young scientist, Dr. Maria Frid, who soon joined our laboratory—turning what was supposed to be a 6‐month visit into a 30‐year collaboration. Together, we revealed that the pulmonary arterial media contains multiple smooth muscle cell subtypes [40], each with distinct responses to hypoxia [41, 42]. This heterogeneity, confirmed decades later by single‐cell sequencing [43, 44], reshaped understanding of vascular remodeling.

We also demonstrated that endothelial cells can undergo endothelial–mesenchymal transition, transforming into smooth muscle–like cells and contributing to intimal thickening [45, 46]—a process now recognized as central to many vascular diseases.

## 5 Fibroblasts, Inflammation, and Complement: A New Frontier

Our studies next turned to fibroblasts, which we found to be unexpectedly dynamic participants in PH pathogenesis. We documented robust fibroblast proliferation and phenotypic diversity under hypoxic conditions [47, 48, 49, 50]. We showed that adventitial remodeling depended on recruitment of circulating mesenchymal precursors—fibrocytes—from a monocyte/macrophage lineage [51, 52].

We discovered that chronic hypoxia induces a persistent, artery‐specific inflammatory microenvironment [53], and that fibroblasts acquire a proinflammatory, epigenetically altered phenotype [54] capable of reprogramming macrophages toward profibrotic behavior [55]. MicroRNA‐124 emerged as a central regulator of fibroblast metabolism and inflammation [56, 57, 58].

Our curiosity about inflammation led us to the complement system, long known for its role in host defense but newly implicated in sterile inflammation [59, 60, 61]. Collaborating with Dr. Thurman and others, we demonstrated that immunoglobulin‐driven complement activation promotes proinflammatory vascular remodeling [62], and that complement factors in plasma correlate with PH severity.

Most recently, we uncovered that intracellular complement—particularly complement factor D (CFD)—drives metabolic and inflammatory reprogramming in PH fibroblasts [63], findings validated by digital spatial profiling of human lesions showing strong CFD upregulation [64].

## 6 Conclusion

From that heartbreaking night in the delivery room to decades of discovery, my journey through pulmonary vascular research has been one of relentless curiosity, collaboration, and hope. The CVP has served as both home and crucible—where ideas became insights and insights became therapies. From identifying leukotrienes in PPHN, to developing the neonatal calf model, to pioneering nitric oxide therapy, each step has been fueled by extraordinary colleagues and trainees whose passion continues to push the boundaries of science.

Together, we have mapped the cellular and molecular terrain of the pulmonary vasculature—uncovering how smooth muscle cells, fibroblasts, inflammation, and complement intersect to shape disease. These discoveries have not only advanced basic science but have transformed patient care and survival.

As I look forward, I am inspired by the next generation of investigators who will carry this work further—armed with ever more powerful tools and the same determination that first took root in that NICU over four decades ago. It has been the privilege of a lifetime to contribute to this field—proof that even the most devastating challenges can, through persistence, imagination, and collaboration, yield to human ingenuity.

## Author Contributions

Dr. Stenmark is the only author and takes full responsibility for the article submitted.

## Ethics Statement

The author has nothing to report.

## Conflicts of Interest

The author declares no conflicts of interest.
